# Editorial: Pain and relationships

**DOI:** 10.3389/fpain.2025.1563825

**Published:** 2025-03-04

**Authors:** Bernie Carter, Line Caes, Joanna McParland

**Affiliations:** ^1^ Faculty of Health, Social Care and Medicine, Edge Hill University, Ormskirk, United Kingdom; ^2^ Department of Psychology, University of Stirling, Stirling, United Kingdom; ^3^ Department of Psychology, Glasgow Caledonian University, Glasgow, United Kingdom

**Keywords:** relationships, social connectedness, persistent pain, therapeutic relationships, resilience, loneliness, mental health

**Editorial on the Research Topic**
Pain and relationships

## Introduction

People are social beings. For the most part, at all stages of their life course, people strive to establish relationships, in order to experience social connectedness (a sense of belonging and being ‘in relation’ with others). Although the relationship between persistent pain and social connectedness is complex and multi-faceted (1) there is good evidence to show that poor social connectedness can amplify pain (2), increases the risk of poor mental health and lower well-being (3–5), and can result in loneliness (3, 6), emotional distress (7), and poorer cognitive function (4). Evidence suggests that psychosocial variables can moderate this negative relationship, such that positivity, resilience and happiness can lessen the experience of pain, even in the face of social isolation (8) and lessen the experience of loneliness (9).

Adding to the complexity, pain not only affects the person in pain but also spreads out to affect those with whom they are in a relationship; this impact is often typified as being negative. Pain can impact friendship networks (10), partner relationships (11, 12), and intimacy, sexual well-being and sexual expectations in relationships (13, 14). Its impact reaches out beyond personal relationships and can affect therapeutic relationships (15).

## Overview of the contributions

This Research Topic brings together a collection of six papers that individually and collectively explore pain, being ‘in relation’, and a variety of different personal and professional relationships.

Whilst in many healthcare papers the pain status of the author(s) is not revealed, in her paper Wilkinson draws attention to herself as a ‘researcher-in-pain’. Considering the ubiquity of persistent pain, it is perhaps surprising that the phenomenon of ‘researcher-in-pain’ has not been previously foregrounded. Wilkinson proposes that ‘researchers-in-pain’ can connect in ways that other researchers cannot do, bringing an embodied responsiveness drawing on their lived insights of pain. This creates a sense of shared vulnerability that has the potential to accommodate ‘pain-friendly’ methods and generate new understandings, inspiring innovative approaches from a position of empathy and understanding.

Putting the person in pain at the centre of treatment requires professionals to closely attend to both what is and is not communicated and to reach out and create connection. In their case report Adachi et al. present how they carefully built a relationship of trust and a therapeutic relationship with Akiko, a patient with fibromyalgia, and deep-rooted negative emotions arising from adverse childhood experience. The therapeutic relationship is achieved through giving Akiko the opportunity express her feelings using non-verbal means (using drawing and being able to hold a ‘towel baby’). The slow, person-centred approach to Akiko and her childhood traumas allowed her to understand her anger and overcome the mistrust and fear engendered by previous unsuccessful medical interventions. By giving attention to the root causes of her pain, Akiko's psychotherapy sessions result in her pain being relieved and being able to become a connected and active person in society.

In another paper considering the therapeutic relationship, Joslin et al. present findings from a scoping review on the importance of therapeutic alliance during physiotherapy treatment for musculoskeletal pain in children. Therapeutic alliance involves a collaborative relationship, an affective bond and agreement on goals and task. Joslin et al.'s narrative synthesis extends the concept of therapeutic alliance within paediatric rehabilitation by identifying the centrality of disciplinary expertise and the importance of being the child's ‘trusted guide’ who can help create a route map for their physiotherapy rehabilitation. They argue that when this occurs resilience is built and children are able to equip themselves with long-term management and problem-solving skills.

Work by Carter et al. explores how young adults with chronic pain and their partners navigate romantic relationships. They define a romantic relationship as a “relationship between two people that extends beyond platonic (just good friends) friendship” (p2), thus widening inclusion of relationships not typically studied in young adults with pain or long-term conditions. The inclusion of partners within this work allows exploration of how dyads navigate their relationship. Whereas previous work has tended to demonstrate negative outcomes, Carter et al. show that despite the challenges and limitations imposed by chronic pain, the relationships of young adults with chronic pain and their partners were characterised more positively, by the ability to be vulnerable with each other, hopefulness and reciprocity.

The relationships between sexual satisfaction and persistent pain are intricate and the evidence base is limited by a focus on biological factors and a preponderance of female participants. In Barr et al.'s study they aimed to examine predictors of sexual satisfaction among male and female adults presenting for chronic pain evaluation. Using a biopsychosocial framework the team undertook chart review and administered retrospective questionnaires (self-reported pain severity, anxiety, depression, sexual functioning, perceived social support). Barr et al. report that approximately 1 out of 5 scored within the clinical range of sexual dissatisfaction. Such findings highlight the need for future sexual satisfaction research and clinical screening to adopt a biopsychosocial approach to research and a greater focus is needed to address protective factors related to sexual satisfaction.

Gonçalves et al. examine the impact of back pain experienced by 350 adolescents (mean age 12.7 ± 1.6 years) attending a public school in Brazil. Using a cross-sectional design they collected self-reported data on the site of back pain (neck, thoracic and low back) and outcomes. Their results show an important association between thoracic pain and low back pain (but not neck pain) with daily activities (missed school classes and interference in physical activities), seeking healthcare, and medication use among school adolescents. They argue that such impacts indicate the need for strategies to prevent and manage back pain in this age group.

## Conclusion

The contributions of these five papers extend understanding of a range of different relationship-centred situations across the lifespan, by prompting reflection on what it is like to be in pain or in-relation with someone in pain, as either a romantic partner, healthcare provider or researcher. Integrating these insights leads to holistic pain management addressing physical, emotional and social elements. The papers explore and reveal the ways in which pain can influence aspects of everyday living as well as indicating how small shifts in practice could be positively influential.

## References

[1] Miller EL. Social connectedness and pain. Pain Manag Nurs. (2023) 24(2):111–2. 10.1016/j.pmn.2023.03.005 37059494

[2] Baumgartner JN Haupt MR Case LK. Chronic pain patients low in social connectedness report higher pain and need deeper pressure for pain relief. Emotion. (2023) 23(8):2156–68. 10.1037/emo0001228 36996174 PMC10544689

[3] Plackett R Hulin J Mukuria C Clowes M Ramsay SE Spencer L Measures of social connectedness in adult populations: a systematic review. BMC Public Health. (2024) 24(1):1–13. 10.1186/s12889-024-20779-0 39639260 PMC11622465

[4] Ritchie CS Patel K Boscardin J Miaskowski C Vranceanu A-M Smith A. Impact of persistent pain on function, cognition, and well-being of older adults. J Am Geriatr Soc. (2023) 71(1):26–35. 10.1111/jgs.18125 36475388 PMC9871006

[5] Tang LH Andreasson KH Thygesen LC Jepsen R Møller A Skou ST. Persistent pain and long-term physical and mental conditions and their association with psychological well-being; data from 10,744 individuals from the lolland-falster health study. J Multimorbidity Comorbidity. (2022) 12:26335565221128712. 10.1177/26335565221128712 PMC9659769 36386291

[6] Forgeron P A Stinson J Birnie K Finley AG Jordan A Qualter P The influence of loneliness on pain outcomes for adolescents: a cross-sectional survey. Canad J Pain. (2024) 8(1):1–13. 10.1080/24740527.2024.2404615 PMC11520531 39473457

[7] Franqueiro AR Yoon J Crago MA Curiel M Wilson JM. The interconnection between social support and emotional distress among individuals with chronic pain: a narrative review. Psychol Res Behav Manag. (2023) 16(null):4389–99. 10.2147/PRBM.S410606 37915959 PMC10617401

[8] Miró J Sánchez-Rodríguez E Nolla MC Costa RM Pais-Ribeiro J Ferreira-Valente A. The role of resilience, happiness, and social support in the psychological function during the late stages of the lockdown in individuals with and without chronic pain. Int J Environ Res Public Health. (2022) 19(11):1–10. 10.3390/ijerph19116708 PMC9180840 35682291

[9] Wilson JM Colebaugh CA Flowers KM Edwards RR Schreiber KL. Profiles of risk and resilience in chronic pain: loneliness, social support, mindfulness, and optimism coming out of the first pandemic year. Pain Med. (2022) 23(12):2010–21. 10.1093/pm/pnac079 35587150 PMC9384018

[10] Bernardes SF Almeida I Forgeron P. Friend or foe? A thematic analysis of adult friendships and chronic pain adjustment. Pain Manag Nurs. (2023) 24(4):375–83. 10.1016/j.pmn.2023.03.003 37037702

[11] Maracci LM Rodrigues AS Knorst JK Salbego RS Ferrazzo VA Liedke GS Does marital status influence TMD-related chronic pain? A cross-sectional study. J Bodyw Mov Ther. (2022) 29:112–6. 10.1016/j.jbmt.2021.12.001 35248258

[12] Tankha H Caño A Dillaway H. “Now I have hope": rebuilding relationships affected by chronic pain. Fam Syst Health. (2020) 38(1):51–6. 10.1037/fsh0000472 32202833 PMC7101054

[13] Myrtveit-Stensrud L Haugstad GK Rème SE Schaller SL Groven KS. “It’s all my fault”: a qualitative study of how heterosexual couples experience living with vulvodynia. Acta Obstet Gynecol Scand. (2023) 102(10):1378–89. 10.1111/aogs.14537 36879489 PMC10540927

[14] Santos-Iglesias P Crump L Henry JL LaChapelle DL Byers ES. The sexual lives of women living with fibromyalgia: a qualitative study. Sex Disabil. (2022) 40(4):669–85. 10.1007/s11195-022-09748-w

[15] Paap D Krops LA Schiphorst Preuper HR Geertzen JHB Dijkstra PU Pool G. Participants’ unspoken thoughts and feelings negatively influence the therapeutic alliance; a qualitative study in a multidisciplinary pain rehabilitation setting. Disabil Rehabil. (2022) 44(18):5090–100. 10.1080/09638288.2021.1924297 33970736

